# ADMIRATION, SKEPTICISM, INDECISIVENESS? ATTITUDES TOWARD AI IN CHINESE MIDDLE-AGED AND OLDER ADULTS

**DOI:** 10.1093/geroni/igad104.3770

**Published:** 2023-12-21

**Authors:** Bobo Hi-po Lau, Eric Ngai Yin Shum, Fan Yin-Fong Lui, Gigi Lam, Alex Pak-Ki Kwok, Chung-Kin Tsang

**Affiliations:** Hong Kong Shue Yan University, Hong Kong, Hong Kong; Hong Kong Shue Yan University, Hong Kong, Hong Kong; Hong Kong Shue Yan University, Hong Kong, Hong Kong; Hong Kong Shue Yan University, Hong Kong, Hong Kong; The Chinese University of Hong Kong, Hong Kong, Hong Kong; Hong Kong Shue Yan University, Hong Kong, Hong Kong

## Abstract

In recent years, AI is increasingly visible in daily lives from chatbots in banking apps and SIRI, and generative AI such as ChatGPT, to more controversial usage concerning facial recognition, online surveillance, and even self-driving cars. Attitudes toward AI may predict acceptance of AI-facilitated applications and the Web 3.0 at-large. In June 2023, we conducted a survey with 750 smartphone users aged 45 years or older (Max = 78) in Hong Kong as a part of a wider effort to elucidate the digital divide post-pandemic. Using principal component analysis with varimax rotation, we found three components from the General Attitude to Artificial Intelligence Scale - Negative, Positive, and Powerful (compared to humans). Subsequently, with latent profile analysis we revealed three profiles: (i) Admiration (18.4%; high on positive and powerful but low on negative); (ii) Skepticism (12.3%; high on negative but low on positive and powerful), and (iii) Indecisiveness (69.3%; moderate on all three components). Admiration was more likely to be male, with higher income, better self-rated health, and greater mobile device proficiency, optimism, innovativeness, but also insecurity with technology, compared to Indecisiveness, and then Skepticism. Corroborating these quantitative findings, AI, or generative AI in specific, were only sparsely featured in a concurrent focus group study on smartphone usage with a similar cohort of smartphone users. Those who mentioned were appreciative of their superior power to provide information beyond traditional search engines; yet apprehension toward associated fraud and deception was also highlighted.

